# Gastrointestinal Pathologies in Pediatric Patients With Cystic Fibrosis Undergoing Endoscopy: A Single-Center Retrospective Review Over 15 Years

**DOI:** 10.7759/cureus.59018

**Published:** 2024-04-25

**Authors:** Nathan A Blaseg, Jacob O Robson, Raza A Patel, Fadi Asfour, John F Pohl

**Affiliations:** 1 Pediatrics, University of Utah Health, Salt Lake City, USA; 2 Pediatric Gastroenterology, University of Utah Health, Salt Lake City, USA; 3 Pediatric Pulmonology, University of Utah Health, Salt Lake City, USA

**Keywords:** celiac disease, eosinophilic esophagitis, endoscopy, gastrointestinal, cystic fibrosis, pediatric

## Abstract

Introduction

Previous studies have demonstrated an increased incidence of gastrointestinal (GI) pathologies, specifically celiac disease (CD) and eosinophilic esophagitis (EoE), in patients with cystic fibrosis (CF). However, there is minimal data available regarding endoscopic findings in pediatric patients with CF and GI mucosal disease.

Methods

A retrospective chart review was performed on patients with CF under 18 years of age who underwent esophagogastroduodenoscopy (EGD) or colonoscopy with biopsy over a 15-year period at our institution. Patient characteristics including assigned sex at birth, CF genetic mutations (if identified), and cystic fibrosis transmembrane conductance regulator (CFTR) modulator use were recorded. Data obtained at the time of biopsy included body mass index (BMI), indication for the procedure, exocrine pancreatic status, visual endoscopic findings, and histologic findings.

Results

A total of 72 patients with CF were included in the study. 24% (n=17) were found to have abnormal endoscopic biopsy results. EoE (13% of all patients, n=9) and CD (6% of all patients, n=4) were the most common GI diagnoses present on endoscopic biopsy. All 3 patients taking CFTR modulator medications at the time of endoscopy had normal biopsy results. Of the 17 patients found to have abnormal pathology results, 14 (82%) were taking proton-pump inhibitor (PPI) medication at the time of endoscopy.

Conclusion

This study highlights the probable increased frequency of GI disease in the pediatric CF population. These findings underscore the importance of maintaining a broad differential diagnosis while considering utilization of endoscopy with biopsy in pediatric patients with CF who have GI symptoms.

## Introduction

Cystic fibrosis (CF) is the most common recessive genetic disorder that is life-threatening for people of European ancestry as well as in other ancestries (such as those of Middle Eastern ancestry), and CF has an incidence of 1:4000 individuals in the United States [1]. Although the life span of patients with CF is increasing, it is still low with the median age of death being 37 years old in the United States in 2020 [2]. While CF is associated with progressive pulmonary disease, patients often experience a multitude of GI signs and symptoms including abdominal pain, nausea, emesis, malnutrition, malabsorption, diarrhea, and constipation due to a multitude of factors [3]. Multicenter studies currently are underway to assess the gastrointestinal (GI) disease burden in patients with CF [3,4]. Because of the associated GI symptoms seen in CF, patients with CF can undergo esophagogastroduodenoscopy (EGD) and/or colonoscopy with an associated biopsy to assess for pathology. Although many pediatric patients with CF undergo such an evaluation, there is a paucity of data regarding the epidemiology of various GI pathologies in the setting of pediatric patients with CF.

Studies have demonstrated altered intestinal microbiome composition and intestinal inflammation in children with CF, potentially predisposing them to inflammatory disorders [5,6]. Interestingly, cystic fibrosis transmembrane conductance regulator (CFTR) modulators used in CF also have been shown to alter intestinal microbiota and have been associated with celiac disease (CD) in small studies [7-11]. Small case series of pediatric patients with CF have described associated CD, eosinophilic esophagitis (EoE), and inflammatory bowel disease [11-16]. Furthermore, studies including both adults and children with CF have demonstrated a potential increased incidence of CD and EoE [5,17].

However, there remains a lack of data regarding the epidemiology of such GI disorders specifically in a large population of pediatric patients with CF, and no large study to date has taken into account CFTR modulator status or specific CFTR genetic mutations in relation to endoscopically proven GI disease. The overall objective of this study was to utilize the electronic medical records of pediatric patients with CF at a single tertiary children’s hospital to determine the presence of associated GI disease found by endoscopic biopsy.

## Materials and methods

This retrospective study was approved by both the Primary Children’s Hospital (PCH) and the University of Utah Institutional Review Boards (IRB 00161562). PCH has a catchment of approximately 1 million children and contains a multidisciplinary pediatric CF clinic with pulmonology, gastroenterology, endocrinology, registered dietitians, and dedicated pharmacists. De-identified electronic medical records of patients under 18 years of age with a diagnosis of CF who had undergone GI endoscopy (EGD and/or colonoscopy) with biopsy at PCH from 2007-2022 were reviewed.

Exclusion criteria included pediatric patients with CF who had not undergone GI endoscopy with associated biopsy or those patients with CF who were age 18 or older at the time of endoscopy. For patients meeting inclusion criteria, the following information was obtained via chart review: age at the time of endoscopy, assigned sex at birth, genetic mutations (if available), and CFTR modulator use status. Data obtained at the time of biopsy include body mass index (BMI), indication for an endoscopic procedure, pancreatic status (pancreatic exocrine insufficiency or exocrine sufficiency), visual endoscopic findings, and histologic findings from endoscopic biopsy. Histologic findings were classified as normal or abnormal based on pathology reports. Histologic findings typically associated with cystic fibrosis, such as crypt dilation with mucosal inspissation, were considered normal [18,19].

Patient characteristics were summarized using descriptive statistics. Categorical data was analyzed using Wilcoxon-Mann-Whitney rank-sum or Pearson’s chi-squared tests where appropriate. A statistically significant difference between groups was defined as a p-value <0.05. Analyses were performed using Microsoft Excel® (Microsoft Corporation, Redmond, USA).

## Results

A total of 72 pediatric patients with CF meeting study inclusion criteria were identified. All patients presented with one clinical GI symptom with the most reported GI symptom prompting endoscopy with biopsy being abdominal pain (23 patients), followed by poor weight gain (21 patients) (Table 1).

**Table 1:** Patient Symptoms Prompting Endoscopy

Indication for Endoscopy	Number of Patients, n (%)
Abdominal Pain	23 (32)
Poor Weight Gain	21 (29)
Dysphagia	8 (11.1)
Vomiting	7 (9.7)
Diarrhea	5 (7)
Reflux	2 (2.8)
Replacing Gastrostomy Tube	2 (2.8)
Hematochezia	2 (2.8)
Evaluation for Varices Due to Cirrhosis	1 (1.4)
Persistent Nausea	1 (1.4)
Total	72 (100)

Of the 72 patients included in the study, 17 (24%) had abnormal biopsy results (Table 2). The overall study patient population was 53% male, with a mean age of 8.1 years and a mean BMI of 16.8 m/kg2. When patients with normal endoscopic biopsy results were compared to those patients with abnormal endoscopic biopsy results, there was no statistically significant difference present in terms of sex, age, or BMI. Pancreatic insufficiency also was not statistically significantly different between both groups, being present in 91% of patients with normal biopsy results and 82% of patients with abnormal biopsy results. The mean dosage of pancreatic enzyme supplementation in those patients with pancreatic insufficiency and abnormal endoscopic biopsy results was 8200 units of lipase/kg of body weight/day. Of the 17 patients found to have abnormal endoscopic biopsy results, 14 (82%) were taking proton pump inhibitor (PPI) medication at the time of endoscopy, with a mean dosage of 0.83 mg/kg/day. PPI dosage was unable to be determined in one patient. Only three patients were taking CFTR modulator medications at the time of endoscopy and biopsy, and all three patients had normal biopsy results. No statistically significant difference could be determined between patients using CFTR modulator medication due to the small number of patients in this group.

**Table 2:** Patient Characteristics CFTR: cystic fibrosis transmembrane conductance regulator

		Biopsy Status		
Measure	All Patients	Normal	Abnormal	P Value
Count, n (%)	72 (100)	55 (76.4)	17 (23.6)	
Sex, n (%)				0.988
Male	38 (52.8)	29 (40.3)	9 (12.5)	
Female	34 (47.2)	26 (36.1)	8 (11.1)	
Age (years)				
Range	<1-17	<1-17	<1-17	
Mean ± SD	8.1 ± 5.7	8.1 ± 5.8	8.2 ± 5.6	0.912
BMI (kg/m2)				
Range	12.9-29.6	13.2-29.6	12.9-21.9	
Mean ± SD	16.8 ± 2.8	17.0 ± 2.9	16.2 ± 2.4	0.280
Pancreatic Status, n (%)				0.327
Sufficient	8 (11.1)	5 (6.9)	3 (4.2)	
Insufficient	64 (88.9)	50 (69.4)	14 (19.4)	
CFTR Modulator, n (%)				
Lumacaftor/Ivacaftor	2 (2.8)	2 (2.8)	0 (0)	
Elexacaftor/Tezacaftor/Ivacaftor	1 (1.4)	1 (1.4)	0 (0)	
None	69 (95.8)	52 (72.2)	17 (23.6)	

Several GI disorders were diagnosed among the 17 patients found to have abnormal endoscopic biopsy results (Table 3). The most common diagnosis was EoE, accounting for 53% of patients with abnormal pathology (nine patients) and for 13% of all patients studied. In all of these patients, EoE was a new diagnosis. CD was diagnosed in 24% of patients with abnormal pathology (four patients) and for 6% of all patients studied. Serum tissue transglutaminase immunoglobulin A (TTG-IgA) was elevated in all four of these patients who had a confirmatory duodenal biopsy for CD, ranging from 28-186 units/mL with a mean of 90.75 units/mL (reference range 0-19 units/mL). Eosinophilic colitis, eosinophilic duodenitis, microscopic colitis, and portal gastropathy were diagnosed in the remaining patients at one patient each. All patients with abnormal biopsies underwent directed treatment of their specific GI disorder based on endoscopic and biopsy findings.

**Table 3:** Gastrointestinal Diagnoses by Biopsy

Diagnosis	Number of Patients (n)	Percentage of Patients with Abnormal Biopsies	Percentage Compared to All Patients (%)
Normal Biopsies	55	-	76%
Abnormal Biopsies	17	-	24%
Eosinophilic Esophagitis	9	53%	13%
Celiac Disease	4	24%	6%
Eosinophilic Colitis	1	6%	1%
Eosinophilic Enteropathy (Duodenitis)	1	6%	1%
Microscopic Colitis	1	6%	1%
Portal Gastropathy	1	6%	1%

Genetic mutations were recorded and compared between those with normal endoscopic biopsy results and those with abnormal biopsy results. The most common mutation was heterozygous p.Phe508del. However, there was a wide variety of reported additional mutations associated with CF. Two patients with heterozygous Q493X mutations were diagnosed with EoE although one patient with normal GI biopsy results also carried this specific mutation. Two patients had heterozygous N1303K mutations, with both patients having abnormal biopsy results. One patient with this specific mutation had EoE, and one patient had eosinophilic colitis. No patients with normal endoscopic biopsy results carried the N1303K mutation. Besides p.Phe508del, there were no other similarities between patients with abnormal biopsy results.

## Discussion

In this study, we demonstrated that 24% of our pediatric patients with CF who underwent GI endoscopy were found to have abnormal biopsy results, with EoE and CD being the most common diagnoses. This rate of this positive diagnostic yield is similar to that of the general pediatric population, with previous studies indicating a rate of abnormal histopathology in pediatric patients undergoing endoscopy and biopsy ranging from 19-44% [20,21]. Overall, these findings highlight the importance of considering GI pathologies, such as EoE or CD, in pediatric patients with CF who may present with GI complaints such as abdominal pain, poor weight gain, dysphagia, gastroesophageal reflux, or a feeding problem [3,5,22].

This study showed that 13% (n=9) of the pediatric patients with CF were diagnosed with EoE. These findings coincide with similar studies in the CF population, as previous reports have indicated EoE to be six times more prevalent in patients with CF [17]. Recent studies have demonstrated an overall prevalence of EoE cases in the general pediatric population in North America to be up to 38.3 cases/100,000 inhabitants. It should be noted that EoE prevalence in Utah, where this study was performed, has been reported to be higher than in any other region of the United States [23,24]. Our current study represents a large pediatric-specific retrospective analysis of EoE incidence in a population of patients with CF, as previous reports have mostly included small case series or populations of predominantly adult patients [13,15,17,22,25].

CD also was found to be relatively common in our study population with it being diagnosed by endoscopy and biopsy in 6% (n=4) of our pediatric patients with CF. These findings corroborate previously published reports which have found an incidence of CD in the pediatric CF population to be as high as 8.6% [7]. While the prevalence of CD varies widely by geographic region, several studies have demonstrated an overall CD prevalence of approximately 1% in the North American pediatric population [26,27]. While the exact mechanism underlying this phenomenon is not completely understood, some studies have implicated the loss of CFTR function in CF leading to an increase in reactive oxygen species within the intestinal microenvironment, leading to increased inflammation, reduced intestinal barrier function, and increased immune reaction against gliadin [16,28,29]. 

Unfortunately, this study included only 3 patients taking CFTR-modulating medications at the time of GI endoscopy and biopsy. However, all 3 patients did have normal pathology results. Previous case series have described patients with CF developing CD while on ivacaftor; however, no large studies have yet been performed to confirm this medication as a risk factor for developing CD [10]. The long-term impact of CFTR modulators on the intestinal microbiome has yet to be fully understood, although previous studies have demonstrated microbiome changes associated with a decrease in intestinal inflammation [10,30]. As CFTR modulator use becomes more commonplace in patients with CF, future studies will be better able to reveal any such association or lack of association with GI disease.

This study represents a relatively large population of pediatric patients with CF and known CFTR genetic mutations in relation to GI disorders. Several associations were noted. Both patients carrying heterozygous N1303K mutations were found to have abnormal biopsy pathology results - one being diagnosed with EoE, and one diagnosed with eosinophilic colitis. A review of the literature did find one previously reported case of a pediatric patient with CF carrying the N1303K mutation who also developed EoE [5]. Furthermore, two patients diagnosed with EoE in our study both carried the heterozygous Q493X mutation, while one patient with normal endoscopic biopsy results carried this same mutation. There are no previously published reports of patients with CF carrying this mutation developing GI pathologies.

This study has several limitations. The relatively small sample size of 72 patients from one tertiary children’s hospital in the United States does create difficulty in terms of statistical analyses and broader conclusions regarding the pediatric CF population as a whole. As an example, we were unable to make any statement about PPI use and possible association between a diagnosis of EoE or CD. A larger sample size would produce more diverse GI endoscopic biopsy and genetic data to allow for clearer associations to be determined in regard to GI disease and specific CFTR mutations. Additionally, there is a potential for selection bias given the fact that this study included only those patients who underwent GI endoscopy, providing little information regarding pediatric CF patients at our center who did not receive endoscopy. There may have been traits apparent to the providers that contributed to the decision to perform endoscopy that were not able to be explored in this study. Lastly, this study did not include an analysis of the intestinal microbiome in these patients which could have been affected by PPI use or CFTR modulator use, and future studies including such information could allow for further elucidation of the underlying mechanisms contributing to the potential for an increased incidence of GI diseases seen in children with CF.

## Conclusions

In summary, this study demonstrates the frequency of abnormal GI endoscopic biopsies present in pediatric patients with CF undergoing endoscopy. We found that nearly one in four of such patients (24%) had abnormal GI biopsy results. These patients were subsequently diagnosed with several GI diseases, with the most common being EoE and CD. These findings underscore the importance of maintaining a broad differential diagnosis and utilizing screening tests such as a TTG-IgA level and endoscopy with biopsy in pediatric patients with CF who have chronic GI complaints.
